# Prevalence and determinants of microbiologically unconfirmed healthcare-associated infections among hospitalized patients in Western Sierra Leone, 2024: a point prevalence survey

**DOI:** 10.1016/j.infpip.2026.100512

**Published:** 2026-01-29

**Authors:** B.D. Fofanah, I.F. Kamara, R.Z. Kamara, R. Musoke, I.B. Nuwagira, R.E. Ngauja, E. Ellie, S.M. Tengbe, S.F.F. Sandy, R.E. Williams, I.A. Bangura, A.R. Mansaray, L. Kabego, S. Lakoh

**Affiliations:** a World Health Organization Country Office, Freetown, Sierra Leone; b Ministry of Health, Freetown, Sierra Leone; c College of Medicine and Allied Health Sciences, University of Sierra Leone, Sierra Leone; d World Health Organization, Regional Office for Africa, Cité du Djoué, Brazzaville P.O. Box 06, Congo

**Keywords:** HAI surveillance, Healthcare-associated infections, Sierra Leone, PLBASI, CLABSI, AMR surveillance, HAIs

## Abstract

**Background:**

Healthcare-associated infections (HAIs) are a major global health problem. Gaps in their detection in low- and middle-income countries may be caused by limited surveillance capacity and lack of contextualized surveillance tools.

**Aim:**

This study aimed to evaluate the prevalence of HAIs and their determinants using contextually adapted HAI case definitions and tools.

**Methods:**

This was a cross-sectional multi-centre point prevalence survey among adults (≥18 years) and children (≥1 month) from 10 hospitals in Western Sierra Leone. The determinants of HAIs were derived using logistic regression analysis and adjusted relative risks with 95% confidence intervals (CIs) reported as a measure of association, and a *P* value ≤0.05 was considered statistically significant.

**Findings:**

Among the 10 hospitals, the mean hospital bed capacity was 109, and the average hand hygiene (HH) compliance was 30.6%. None of the hospitals had a functional bacteriology laboratory. Of the 319 eligible patients, 40 (12.5%) acquired HAIs, with 31 (77.5%) having suspected bloodstream infections. Cigarette smoking (adjusted odds ratio [aOR]: 3.764, 95% CI: 1.07–13.13, *P*: 0.038), leucopenia (aOR: 0.137, 95% CI: 0.019–1.003, *P*: 0.05), blood transfusion (aOR: 0.309, 95% CI: 0.126–0.759, *P*: 0.01), and use of oxygen apparatus (aOR: 0.260, 95% CI: 0.084–0.805, *P*: 0.02) were shown to be predictors of HAIs.

**Conclusion:**

There was a high prevalence of microbiologically unconfirmed HAIs with patient-related factors and service delivery interventions as independent predictors. Fundamental gaps are low HH compliance and limited laboratory capacity to support HAI surveillance. We recommend sustained HH improvement initiatives, strengthening bacteriology capacity for HAI detection, and periodic point prevalence surveys on HAIs as an entry point.

## Introduction

Healthcare-associated infections (HAIs), including those caused by resistant microbes, are a serious threat to global health [1,2]. HAIs are infections that occur during care in a hospital or other healthcare settings [3,4] and are divided into two broad categories. Device-associated HAIs include catheter-associated urinary tract infections (CAUTIs), central line–associated bloodstream infections (CLABSIs), and ventilator-associated pneumonia (VAP). Non-device-associated HAIs include surgical site infections (SSIs) and healthcare-associated pneumonia (HAP) [5].

HAIs are a major global health problem affecting millions of people globally [6]. There is a disproportionately high burden of HAIs in low- and middle-income countries (LMICSs) with a pooled prevalence of 15.5%, which is about three folds higher than in high-income countries [5,6]. Sub-Saharan Africa (SSA) has an estimated pooled prevalence of 12.9% [7]. A recent systematic review of HAIs in SSA reported studies with prevalence rates as high as 90% among hospitalized patients [8]. These infections if not prevented or treated, pose a huge impact on the health and well-being of the population, increasing the spread of resistant infections and leading to excess morbidity and mortality [[9], [10], [11]]. The crude excess mortality is 18.5% for CAUTIs, 23.6% for CLABSIs, and 29.3% for VAP in adult patients [5].

Despite the substantial burden caused by HAIs, gaps in their detection in LMICs remain a huge challenge [12]. This may be largely due to limited surveillance capacity and a lack of contextual surveillance tools such as context-based case definitions. Therefore, effective prevention and control of HAIs requires context-specific strategies, particularly in SSA, where existing health infrastructure is severely limited [11]. There are currently no national representative data on HAIs to inform public health interventions in Sierra Leone. Small studies have reported a high prevalence of HAIs, but most have been limited to only one form of HAI. The prevalence of SSIs in Sierra Leone varies among different surgical patient populations, ranging from 1.2% to 11.5% [[13], [14], [15], [16], [17]], with CAUTIs reported in 14.8% of catheterized patients in two hospitals in Sierra Leone [18]. There are no data on CLABSIs and VAP, which further reinforces the limitation in the evidence that is needed to prevent and control HAIs. Hence, a point prevalence survey will provide systematic and empirical data for a comprehensive characterization of the scope of HAIs to offer data-driven recommendations for informed measures to be taken by the government and health development partners towards HAI prevention and surveillance.

In this study, we primarily aimed to evaluate the prevalence of HAIs and their determinants using contextually adapted HAI case definitions and tools, thereby providing evidence to guide the design and implementation of strategies to reduce the HAI burden in the country. Additionally, we described hospital readiness status for routine HAI surveillance within a point prevalence survey framework.

## Methods

### Study design

We used a cross-sectional design by record review of routinely collected patient data through a multi-centre point prevalence survey.

### Study setting

Sierra Leone is divided into five geographic regions. The Western Area is the most populated region of Sierra Leone and includes the capital city, Freetown. According to the 2015 population census, Sierra Leone has approximately 7.2 million people, of which 22% (1.5 million) reside in the Western Area [19]. About 42% of the 16,350 healthcare workers employed by the government are in the Western Area [20]. The public health system in Sierra Leone is divided into three levels of care, including primary, secondary, and tertiary care, across the five regions, with the Western Area having the highest number of hospitals. Tertiary hospitals are referral hospitals that provide specialist care services, while secondary hospitals provide basic surgical and medical services. Ten acute care hospitals in Western Sierra Leone comprising a total bed capacity of 1096 were selected to participate in the survey, including 8 public and 2 private hospitals, which are either providing secondary or tertiary care services (Table I). These facilities were selected because they provide acute care services, have links to many primary care facilities, and have infection prevention control (IPC) programmes with dedicated leadership to support HAI surveillance activities in the hospitals.

**Table I:** Hospital characteristics and IPC status of ten acute care hospitals in Freetown in 2024

Variable	Frequency (*N* = 10)	Percentage (%)
Ownership		
Private	2	20
Public	8	80
Hospital type		
Secondary	6	60
Tertiary	4	40
Number of hospital beds (mean ± SD)	109 ± 78.7	
<50	5	50
50–200	4	40
>200	1	10
Number of ICU/HDU beds [median (IQR)]	7.5 (0.0–12.25)	
<5	4	40
5–10	3	30
>10	3	30
Total included wards/units (mean ± SD)	71.6 + 63.9	
Does hospital work plan include IPC activity?		
Yes	3	30
No	7	70
Does the IPC programme have work plan approved by the hospital administration?		
Yes	3	30
No	7	70
If yes, does the IPC work plan include HAI surveillance?	3	100
The hospital participates in the previous HAI surveillance		
None	7	70.0
SSI	3	30.0
Is there a functional bacteriology lab to support HAI surveillance?		
No	10	100
Is hand hygiene compliance monitored in the last 3 months?		
Yes	7	70.0
No	3	30.0
Average hand hygiene compliance score for the hospital	30.6%	

Abbreviations: HAI: healthcare-associated infection; HDU: high-dependency unit; IPC: infection prevention and control; ICU: intensive care unit; IQR: interquartile range; SD: standard deviation.

### Study inclusion and exclusion criteria

#### Inclusion criteria

We collected data from all adults (≥18 years) and children (1 month–17 years) admitted to ten acute care hospitals in February 2024. We included all patients who were admitted to the hospital at or before 8 a.m. on the day of the survey and who had not been discharged at the time of the survey. The following wards were included: medical, surgical, paediatric, intensive care unit, high-dependency unit, maternity, emergency, and mixed wards.

#### Exclusion criteria

We excluded patients admitted to accident and emergency wards, special care baby units, outpatient departments, dialysis units, infectious disease wards, and ophthalmology wards because of the rapid turnover of admissions in these wards. Patients in long-term care units or those undergoing same-day treatment or surgery and patients who received dialysis on an outpatient basis were excluded from the study.

### Case definitions

As part of our preliminary work to develop an HAI surveillance strategy for Sierra Leone, we developed a national protocol for a point prevalence survey of HAIs by adapting the case definitions of HAIs from the European Centre for Disease Prevention and Control contextually [21] and the Sierra Leone National IPC Guidelines on HAI surveillance [22]. These adopted case definitions are in line with the recent World Health Organization (WHO) practical guide for the surveillance of HAIs, which suggests similar classification of HAIs, especially in settings with limited resources, such as microbiologically confirmed, not microbiologically confirmed, radiologically confirmed, and suspected cases [23].

We added peripheral line–associated bloodstream infections (PLABSIs) to define bloodstream infections associated with peripheral line cannulation. PLABSIs have been described in the literature and may be a public health problem in our setting, but it has not yet been characterized by international guidelines [24,25]. Because most hospitals in Sierra Leone do not have a microbiology laboratory, the case definitions for CAUTIs and CLABSIs/PLABSIs were divided into microbiologically confirmed and microbiologically unconfirmed categories to increase detection coverage. Similarly, HAP and VAP were divided into radiologically confirmed and radiologically unconfirmed categories to extend surveillance to hospitals without radiology facilities. We did not modify the case definition for SSIs.

Microbiologically unconfirmed CLABSIs/PLABSIs were defined as an infection in a patient in whom an intravascular catheter (peripheral line or central line) was in place for >2 calendar days during the present admission and

− a suspected bloodstream infection with at least one of the following symptoms: fever (>38 °C), chills, or hypotension (for patients ≤1 year: fever, hypothermia, apnoea, or bradycardia) *AND*
− no blood culture done *AND*
− no apparent infection at another site *AND*
− physician-instituted treatment for sepsis

We defined microbiologically unconfirmed CAUTIs as an infection in a patient with an indwelling urinary catheter in place for >2 days during the present admission and

− with at least two of the following symptoms: fever (>38 °C), urgency, frequency, suprapubic or flank pain/tenderness, altered mental status in the elderly (>70 years), dysuria (or in children: a crying baby when passing urine); *AND*
− at least one of the following symptoms: positive dipstick urine (with positive leukocytes and or nitrite), pyuria (≥10 white blood cells/mL), organisms of unspun urine, ≥2 urine cultures from the same uropathogen with ≥10^2^ colonies/mL, physician diagnosis, or treatment of urinary tract infection.

### Data collection instruments

We recruited and trained research nurses and data collectors, including IPC focal persons and surveillance officers. Five teams were established, each consisting of a supervisor and three data collectors. All the staff members selected for data collection are competent in HAI surveillance and data collection. The team supervisor is experienced with health-related surveys and HAI surveillance. The supervisor coordinates the team’s activities within the assigned hospital, verifying that the researchers are filling out the questionnaires completely and correctly. The team supervisors also ensured that the teams had all the necessary materials and maintained data quality. Data were collected from patients’ records.

All data tools were pretested by the study team before the survey. The first was the hospital system form, which assessed the hospital characteristics, IPC programmes, and system for HAI surveillance and prevention (Appendix B). The second tool (ward form) assessed indicators of the ward characteristics, including hand hygiene (HH) and sanitation facilities, ward rooms and bed occupancy (Appendix C). The third tool is a questionnaire (patient form) that was used to collect basic demographic information for each participant as well as the potential risk factors and the trigger questions for the different types of HAIs: CLABSIs, PLABSIs, CAUTIs, SSIs, VAP, and HAP (Appendix D). Each patient was assigned a unique identification number, and clinicians in the respective hospitals were informed about patients with features of HAIs for further evaluation and management.

We did not collect specimens for any investigation or send patients for a chest X-ray but recorded the microbiology, WBC count, and chest X-ray findings where they were available.

### Data entry and statistical analysis

The data were entered manually by two experienced data entry clerks into an Excel database designed specifically for the survey. The data were validated and coded by data entry clerks and the study investigators before being transferred to statistical software for analysis.

All analysis was done using Statistical Package for the Social Sciences (SPSS; IBM Corporation, Armonk, New York, USA)version 29. Categorical variables and HAI outcomes were described using frequencies with the corresponding percentages and their 95% confidence intervals (CIs). In this study, the primary outcome of interest was the prevalence of HAIs. The independent variables explored are the sociodemographic and clinical variables.

Univariate regression was conducted to estimate the unadjusted risk ratios and 95% CIs for each independent variable. Variables with a *P* value <0.2 on univariate analysis were included in the multi-variate analysis. A multi-variate regression analysis was done to identify predictors that are independently associated with HAI outcomes. Adjusted relative risks with 95% CIs were reported as a measure of association, and a *P* value ≤0.05 was considered statistically significant in the multi-variate analysis.

## Results

### Hospital characteristics and IPC status

Of the 10 hospitals assessed, eight (80%) were public health facilities and six (60%) were classified as secondary-level care facilities. The mean hospital bed capacity was 109. Only three (30%) hospitals had IPC activities in their work plan, and all included HAI surveillance. However, none of the hospitals that participated in HAI surveillance had a functional bacteriology laboratory to support HAIs or antimicrobial resistance (AMR) surveillance at the time of the survey. Additionally, only one tertiary hospital has antibiotic treatment guidelines. HH compliance was monitored in seven (70%) hospitals, with an average compliance score of 30.6% (Table I).

### Patient characteristics and risk factors of HAIs

Among the 319 patients enrolled in this study, 199 (62.4%) were female. The mean age of participants is 37.9 years (standard deviation = 84.6). Comorbidities were reported in 87 (27.2%) participants: diabetes (9.7%), HIV (8.2%), and chronic lung diseases (8.2%). About 86.5% of the patients received antibiotics. Other HAI risk factors were burns (9.4%), blood transfusion (8.8%), loss of consciousness (8.2%), and intranasal oxygen use (4.7%) (Table II).

**Table II:** Patients’ characteristics and risk factors for HAIs among inpatients from 10 acute care hospitals in Freetown in 2024

Characteristics	Frequency (*N* = 319)	Percentage (%)
*Sociodemographic*		
Age (years) (mean ± SD)	32.9 (±23.9)	
<1	23	7.2
1–14	65	20.4
15-34	85	26.6
35-64	111	34.8
>64	35	11.0
Gender		
Male	120	37.6
Female	199	62.4
Smoking	12	3.8
***Antibiotic use***	276	86.5
Treatment	266	83.3
Prophylaxis	9	2.8
Prophylaxis & treatment	1	0.3
***Comorbidity***	87	27.2
Diabetes	31	9.7
Stroke/Paraplegia	19	6.0
Chronic lung disease	26	8.2
Obesity	11	3.4
***Other risk factors***		
Malnutrition	5	1.6
Leucopenia	4	1.3
HIV	26	8.2
Burns	30	9.4
Immobility	13	4.1
Loss of consciousness	26	8.2
Surgical drain	14	4.4
Steroid use	3	0.9
Blood transfusion	28	8.8
Use of oxygen	15	4.7

HAI: healthcare-associated infection; SD: standard deviation.

### Prevalence and determinants of HAIs

Results from 319 patients show that 40 (12.5%) had at least one HAI, with the majority, 77.5% (31/40), of them having microbiologically unconfirmed PLABSIs, 20% (8/40) of HAIs were superficial SSIs, and 2.5% (1/40) were CAUTIs (Table III). On the multi-variable analysis, cigarette smoking (adjusted odds ratio [aOR]: 3.764, 95% CI: 1.07–13.13, P: 0.038), leucopenia (aOR: 0.137, 95% CI: 0.019–1.003, P: 0.05), blood transfusion (aOR: 0.309, 95% CI: 0.126–0.759, P: 0.01), and use of oxygen (aOR: 0.260, 95% CI: 0.084–0.805, P: 0.02) were found to be independent predictors of HAIs (Table IV).

**Table III:** Prevalence of HAIs among inpatients from 10 acute care hospitals in Freetown in 2024

Characteristics	N	%
HAI	40	12.5
Type of HAI (*N* = 40)		
Microbiologically unconfirmed CAUTI	1	2.5
Microbiologically unconfirmed PLABSI	31	77.5
Superficial SSI	8	20

HAI: healthcare associated infection; CAUTI: catheter-associated urinary tract infection; PLABSI: peripheral line–associated blood stream infection.

**Table IV:** Predictors of HAIs among inpatients from ten acute care hospitals in Western Sierra Leone

Variable	HAI		OR (95% CI)	*P* value	aOR (95% CI)	*P* value
	Yes, *N* (%)^a^ *40 (12.5)*	*No*, *N (%)*^b^ *279 (86%)*				
**Age (years)**				0.908		0.910
<1	2 (5.0)	21 (7.5)	0.74 (0.12–4.40)		1.355 (0.2–8.0)	0.739
1–14	8 (20.0)	57 (20.4)	1.09 (0.30–3.90)		0.919 (0.3–3.3)	0.897
15–34	13 (32.5)	72 (25.8)	1.40 (0.42–4.62)		0.715 (0.2–2.4)	0.582
35–64	13 (32.5)	98 (35.1)	1.03 (0.31–3.38)		0.973 (0.3–3.2)	0.964
>64	4 (10.0)	31 (11.1)	Ref		1	
**Gender**				0.441		0.442
Male	13 (32.5)	107 (38.4)	0.78 (0.4–1.49)		1.331 (0.6–2.7)	
Female	27 (67.5)	172 (61.6)	Ref		1	
**Smoking**				0.027∗		0.038∗
No	38 (92.7)	269 (96.8)	0.266 (0.08–0.93)		3.76 (1.1–13.1)	
Yes	3 (7.3)	9 (3.2)	Ref		1	
**Diabetes**				0.076		0.082
No	33 (82.5)	255 (91.4)	0.566 (0.256–1.249)		0.444 (0.17–1.1)	
Yes	7 (17.5)	24 (8.6)	Ref			
**Stroke**				0.248		0.511
No	36 (90.0)	264 (94.6)	0.689 (0.234–2.030)		0.649 (0.18–2.36)	
Yes	4 (10.0)	15 (5.4)	Ref		1	
**Chronic lung disease**			0.163		0.219	
No	39 (97.5)	254 (91.0)	3.250 (0.46–22.75)		3.57 (0.47–27.2)	
Yes	1 (2.5)	25 (9.0)	Ref		1	
**Obesity**				.503		0.508
No	39 (97.5)	275 (98.6)	0.635 (0.175–2.312)		0.588 (0.12–2.8)	
Yes	1 (2.5)	9 (1.4)	Ref		1	
**Malnutrition**			0.612		0.572	
No	39 (97.5)	273 (98.6)	0.583 (0.098–3.453)		0.527 (0.05–4.9)	
Yes	1 (2.5)	4 (1.4)	Ref		1	
**Leucopenia**			0.023∗		0.050	
No	38 (95.0)	277 (99.3)	0.241 (0.087–0.672)		0.137 (0.01–1.0)	
Yes	2 (5.0)	2 (0.7)	Ref		1	
**HIV status**			0.436		0.504	
Nonreactive	38 (95.0)	255 (91.4)	1.686 (0.431–6.596)		1.660 (0.38–7.3)	
Reactive	2 (5.0)	24 (8.6)	Ref		1	
**Burns**				0.131		0.164
No	39 (97.5)	250 (89.6)	3.803 (0.54–26.8)		4.210 (0.56–31.9)	
Yes	1 (2.5)	29 (10.4)	Ref		1	
**Immobility**			0.590		0.707	
No	39 (97.5)	267 (95.7)	1.430 (0.214–9.577)		1.489 (0.19–11.9)	
Yes	1 (2.5)	12 (4.3)	Ref		1	
**Loss of consciousness**			0.044∗		0.999	
No	40 (100.0)	253 (90.7)	0.872 (0.834–0.911)		5.72e10 (0.0- ∞)	
Yes	0 (0.0)	26 (9.3)	Ref		1	
**Surgical drain**			0.840		0.767	
No	38 (95.0)	267 (95.7)	0.872 (0.234–3.255)		0.792 (0.17–3.69)	
Yes	2 (5.0)	12 (4.3)	Ref		1	
**Steroid use**			0.510		0.999	
No	40 (100.0)	276 (98.9)	0.881 (0.846–0.918)		2.17 (0.000)	
Yes	0 (0.0)	3 (1.1)	Ref		1	
**Blood transfusion**			0.007∗		0.010∗	
No	32 (80.0)	259 (92.8)	0.385 (0.126–0.830)		0.309 (0.13–0.8)	
Yes	8 (20.0)	20 (7.2)	Ref		1	
**Use of oxygen**			0.013∗		0.020∗	
No	35 (87.5)	269 (96.4)	1.339 (0.926–1.903)		0.260 (0.08–0.8)	
Yes	5 (12.5)	10 (3.6)	Ref		1	

aOR: adjusted odds ratio; CI: confidence interval; HAI: healthcare-associated infection.

a Row percentage for each of Yes, HAI defined.

b Row percentage for each of No HAI defined.

## Discussions

We described the prevalence, determinants, and status of surveillance of HAIs in ten hospitals in Western Sierra Leone, highlighting four key findings. First, only three out of ten hospitals reported incorporating IPC activities into their work plans or performing HAI surveillance. Second, none of the ten hospitals had a fully functional bacteriology laboratory to support HAI surveillance, treatment, and prevention. Third, HH compliance is low, with an average compliance score of 31% for the ten hospitals. Finally, there was a high prevalence of HAIs (12.5%) and factors such as cigarette smoking, leucopenia, blood transfusion and oxygen use were independent predictors of HAIs.

The high prevalence of HAIs in our study is similar to the reported prevalence of 12.9% for the African region including that of 10.9% from Tunisia, but lower than country-specific prevalence of 19.4% in Ethiopia, 14.3% in Nigeria and 32.0% in Rwanda, underscoring the need for a coherent regional approach to strengthen HAI surveillance, treatment and prevention in Africa [7,[26], [27], [28], [29]]. Unlike in Africa, the prevalence of HAIs is declining in the Global North [30]. Geographical differences in the prevalence of HAIs can be explored to share experiences, best practices, and expertise through various North–South collaborations.

We contextualized the case definitions to include the previously described but rarely evaluated PLABSIs [25,31]. This deliberate effort to add PLABSIs into the HAI surveillance system is innovative and based on the fact that peripheral intravenous cannulation is a common practice in both formal and informal healthcare settings in Sierra Leone. More importantly, microbiologically unconfirmed PLABSIs were the most common HAIs reported in this study.

Unfortunately, laboratory capacity is limited to support HAI surveillance, treatment, and prevention as none of the ten hospitals have a fully functional bacteriology laboratory. As described in a previous study, limited laboratory support for microbiological methods is a perennial problem in Sierra Leone and continues to hamper service delivery, including HAI and AMR surveillance [32]. Although efforts are underway to establish these structures in selected hospitals by renovation and equipping of six health facility laboratories in Sierra Leone through the Fleming Fund Country Grant on AMR [33], more efforts are needed to embed bacteriology services to support integrated HAI and AMR surveillance in hospitals with periodic point prevalence surveys on HAIs as an entry point. The availability of treatment guidelines in one major hospital (Connaught Hospital) was promising as our recent national point prevalence survey on antibiotic use reported poor adherence to antibiotic treatment guidelines nationwide [34].

Our study reported a low HH compliance rate of 31% in hospitals in Western Sierra Leone, which is similar to previous reports [35]. Studies have previously described declining HH compliance and HH promotion in some hospitals in Western Sierra Leone following the end of the acute phase of the COVID-19 pandemic [36,37]. Known barriers in delivering IPC services, such as HH, could explain the low HH compliance rate [38]. In the era of emerging and re-emerging infectious diseases, we need sustained improvements in HH compliance and promotion services.

We reported biomedical-related factors (leucopenia and diabetes), patient behaviour (cigarette smoking), and service delivery interventions (blood transfusion, also related to the peripheral line, and oxygen use) as independent predictors of HAIs. This could be explained by the severity of the disease conditions and delayed care seeking, warranting education of the population on the consequences of delayed care seeking. These observations were similar to other studies, except that malnutrition, immunosuppression, length of stay, and re-admission were considered predictors for HAIs in other studies [27,39,40].

The strength of this study is reflected in its scope, contextual adaptation, and regional approach as we assessed all forms of HAIs using contextually adapted case definitions and tools in Western Sierra Leone. It was feasible to use the locally adapted case definition and tools for HAI surveillance in low-resource settings. This study provides contextual justification for the recent WHO guidance on HAI surveillance, suggesting the adoption and reporting of HAIs based on simplified case definitions not requiring a high-level laboratory, diagnostic, imaging, and epidemiological capacity that may not be achievable in low-resource settings like Sierra Leone [25]. Furthermore, the data from this assessment have been used to strengthen the evidence to develop the first HAI surveillance strategy, thus contributing to improving Sierra Leone’s IPC performance and Joint External Evaluation scores [41,42]. Finally, we adhered to the Strengthening the Reporting of Observational Studies in Epidemiology guidelines for the conduct and reporting of observational studies [43].

This study is limited by the fact that it was a point prevalence survey. Therefore, we did not evaluate the impact of HAIs on mortality, health-related quality of life, economic losses, and length of hospital stay, which could be done in future studies. The possibility of having more patients admitted at least less than 24 h before the survey day may have resulted in a likely underestimated prevalence. We could not get microbiological cultures or imaging reports on patients with HAIs because of limited resources and the fact that most hospitals do not offer these services. We used the laboratory or imaging data only when available. Nonetheless, the data collected from different hospitals were comparable due to the consistency of the sampling methods.

In conclusion, we report a high prevalence of microbiologically unconfirmed HAIs in Western Sierra Leone based on contextually adapted HAI case definitions. Patient-related factors and service delivery interventions were independent predictors of HAIs. Fundamental gaps are low HH compliance and limited laboratory capacity to support HAI surveillance, treatment, and prevention. We recommend regular point prevalence surveys on HAIs as an entry point, sustained HH improvement initiatives, and strengthening of bacteriology capacity to support HAI and AMR surveillance.

## CRediT authorship contribution statement

**B.D. Fofanah:** Conceptualization, Data curation, Formal analysis, Investigation, Methodology, Project administration, Resources, Validation, Visualization, Writing – original draft, Writing – review & editing. **I.F. Kamara:** Conceptualization, Investigation, Methodology, Visualization, Writing – review & editing. **R.Z. Kamara:** Conceptualization, Investigation, Methodology, Writing – review & editing. **R. Musoke:** Supervision, Writing – review & editing, Funding acquisition. **I.B. Nuwagira:** Supervision, Writing – review & editing, Funding acquisition. **R.E. Ngauja:** Investigation, Validation, Visualization. **E. Ellie:** Investigation, Validation, Visualization, Writing – review & editing. **S.M. Tengbe:** Methodology, Visualization, Writing – review & editing. **S.F.F. Sandy:** Data curation, Formal analysis, Methodology. **R.E. Williams:** Investigation, Validation, Visualization. **I.A. Bangura:** Investigation, Validation, Visualization, Writing – review & editing. **A.R. Mansaray:** Validation, Visualization, Writing – review & editing. **L. Kabego:** Methodology, Validation, Visualization, Writing – review & editing. **S. Lakoh:** Conceptualization, Data curation, Formal analysis, Investigation, Methodology, Project administration, Supervision, Writing – original draft, Writing – review & editing.

## Availability of data

The datasets for this study will be available upon reasonable request from the corresponding author.

## Ethics approval

Ethical approval was sought from the Sierra Leone Ethics and Scientific Review Board. This study was a component of IPC programme implementation and is a routine programmatic evaluation, collecting secondary data from case records. The data collection methods do not pose any risk to the survey participants.

## Funding sources

The Government of the United States, represented by the United States Centers for Disease Control and Prevention (US CDC), contributed a designated fund for the development of a national HAI Surveillance Strategy for Sierra Leone through the CDC CoAg portfolio implemented by the World Health Organization Sierra Leone under the award number 74018.

## Conflict of interest statement

The authors declare that they have no competing interests. The funders had no role in the design of the study.

## Appendices

The supporting documents used in this study are available in the Supplementary File 1 which contains Appendix A (healthcare-associated infection case definitions), Appendix B (hospital system form), Appendix C (ward form), and Appendix D (patient form).
