# Supplementary material for: Prevalence and determinants of microbiologically unconfirmed healthcare-associated infections among hospitalized patients in Western Sierra Leone, 2024: a point prevalence survey
Source: Infect Prev Pract. 2026 Jan 29;8(2):100512. doi: 10.1016/j.infpip.2026.100512 (PMC13080673; doi:10.1016/j.infpip.2026.100512)
Supplement: Multimedia component 1 [file mmc1.docx]

**APPENDIX A**

**HAI CASE DEFINITIONS**

| **Case definition for CAUTI** | | |
| --- | --- | --- |
| **Eligibility** | Patient had an indwelling urinary catheter in place for >2 calendar days, with day of device placement being Day 1, and the catheter was in place or removed within 24 hours. | |
| **Case classification** | **Microbiologically confirmed**  **symptomatic UTI** | **Microbiologically unconfirmed**  **symptomatic UTI** |
| **Criteria** | **Patient must meet criteria 1, and 2**   1. Patient has at least one (1) of the following (no other cause):  - fever (>38^o^C) - urgency - frequency - suprapubic or flank pain/tenderness - Altered mental status in elderly (>70 years) - Dysuria - In children: a crying baby when passing urine   ***AND***   1. Positive urine culture (≤2 species)/ml)*   Urine samples  *Mid-stream urine for adult and children (>10^5^)  *Catheter urine under sterile procedure when necessary (10^3^) | **Patient must meet criteria 1, and 2**   1. Patient has at least two (2) of the following  - fever (>38oC) - urgency - frequency - suprapubic or flank pain/tenderness - Altered mental status in elderly (>70 years) - Dysuria - In children: a crying baby when passing urine   ***AND***   1. At least one of the following  - positive dipstick urine* - pyuria (≥10 white blood cells/ml) - Organisms/gram of unspun urine - ≥2 urine cultures same uropathogen ≥10^2^ organisms/ml - *physician diagnosis of UTI* - *physician treatment of UTI*   *Dipstick urine test that show positive leukocytes and or nitrite |
| **Comment** | Note: Do not report asymptomatic bacteriuria | |

| **Case definition for CLABSI/PLABSI** | | |
| --- | --- | --- |
| **Eligibility** | A patient in which there been an intravascular catheter was in place for >2 calendar days on the date of event, or on the day of removal, or within 24hrs of removal. | |
| **Case classification** | **Microbiologically confirmed BSI** | **Microbiologically unconfirmed BSI (Clinical Sepsis)** |
| **Criteria** | Patient must meet criteria 1 or 2  1: Laboratory-confirmed bloodstream infection with recognized pathogen cultured from 1 or more blood samples^@^  **OR**  2. Patient meets at least the following (2a and 2b)   1. At least 1 of the following signs or symptoms:  - fever - chills, or - hypotension - for patients ≤1 year: fever, hypothermia, apnoea, or bradycardia   **and**   1. Common skin contaminant is cultured from 2 or more blood cultures drawn on separate occasions^#^ | Patient must meet criteria 1a, 1b, and 1c,  1. Laboratory-unconfirmed bloodstream infection with the following   1. At least one of the following signs or symptoms:  - Fever - chills, or - hypotension - for patients ≤1 year: fever, hypothermia, apnoea, or bradycardia  1. Blood culture not done or negative 2. No apparent infection at another site or Physician institutes treatment for sepsis |
| **Comment** | Note:  *^@^Enterococcus spp., E. coli, Pseudomonas spp., Klebsiella spp., Acinetobacter spp., Candida spp., Staphylococcus aureus*  *^#^Coagulase Negative Staphylococci (CONS), Diphtheroids, Bacillus* | |

| **Case definition for HAP/VAP** | | |
| --- | --- | --- |
| **Eligibility** | Patients who received mechanical ventilation or with history of previous hospitalization for >2 calendar days on the date of the event (with the day of ventilator placement being day 1) or on the day of removal, or within 24hrs of removal. | |
| **Case classification** | **Radiologically confirmed HAP or VAP** | **Radiologically unconfirmed HAP or VAP** |
| **Criteria** | Patient must meet criteria 1, 2, 3 and/or 4   1. At least ONE of the general criteria **(can be different for children/infants)*** 2. Fever > 38◦C 3. Leukopenia (<4000 WBC/mm3) or Leucocytosis (≥12,000 WBC/mm3). *For children ( 4. Altered mental status (≥ 70 years)   ***AND***   1. At least ONE pulmonary clinical criterion 2. New onset of purulent sputum, or change in character of sputum, or increased respiratory secretions, or increase suctioning requirements 3. Cough, 4. dyspnoea 5. tachypnoea 6. Rales/crepitations 7. bronchial breathing 8. Worsening gas exchange   ***AND***   1. **ONE radiological criterion** 2. Patient with underlying cardiac or pulmonary diseases with two or more chest x-rays or 3. patient without underlying cardiac or pulmonary diseases with at least one chest X-ray; With  - new or progressive and persistent infiltrates, ***or*** - consolidation, ***or*** - cavitation   **AND (Optional microbiologic criterion – not necessary for confirmation of HAI)**   1. ONE Optional microbiologic criterion  - Positive quantitative cultures of lower respiratory tract specimen (e.g., bronchoalveolar lavage or protected specimen brushing) - Positive blood culture not related to other source of infections - Positive pleural fluid culture - Positive histopathologic exam | Patient must meet criteria 1, and 2   1. At least ONE of the general criteria (can be different for children/infants)* 2. Fever > 38◦C 3. Leukopenia (<4000 WBC/mm3) or Leucocytosis (≥12,000 WBC/mm3). *For children ( 4. Altered mental status (≥ 70 years)   ***AND***   1. At least ONE pulmonary clinical criterion 2. New onset of purulent sputum, or change in character of sputum, or increased respiratory secretions, or increase suctioning requirements 3. Cough, 4. Dyspnoea 5. Tachypnoea 6. Rales/crepitations 7. bronchial breathing 8. Worsening gas exchange |
| **Comment** | In the absence of imaging facilities, a patient that fulfils both the general and pulmonary clinical criteria should be considered to have radiologically unconfirmed HAP or VAP | |

| **Case definition for SSI** | | |
| --- | --- | --- |
| **Eligibility** | A patient within 30 days of the surgical procedure with the following observed or reported.  Excluding the following: Stitch *abscess, Localized stab wound, Infection from the circumcision site of the newborn, Infected episiotomy wound, Infected burn wound* | |
| **Case classification** | **Surgical site infection involving superficial incision, deep incision, and organ/space** | |
| **Criteria** | Patient must meet criteria 1 or 2 or 3   1. A purulent (pus) discharge in, or coming from the wound (including evidence of an abscess)   **OR**   1. Evidence of fever with painful, spreading erythema surrounding the surgical site   **OR**   1. Any reopening of the surgical wound |  |
| **Comment** | Note: | |

**APPENDIX B**

**SIERRA LEONE NATIONAL HEALTHCARE ASSOCIATED INFECTIONS SURVEILLANCE**

**HOSPITAL SYSTEM DATA FORM**

| ***Hospital name: …………………………………………….*** | ***Hospital code: …………………………….*** |
| --- | --- |
| ***Hospital size (total number of beds): ….……………*** | ***Survey date: ……………………………….*** |
| ***Number of ICU/HDU beds: ……………………………..*** | ***Number of acute care beds: ……………*** |
| ***Exclusion of wards for PPS? No Yes***  ***If yes, please specify which ward types were excluded ………………………………………………….***  ***……………………………………………………………………………………………………………………….*** | |
| ***Total number of beds in included wards: ……………*** | ***Total number of patients in PPS: ……..*** |
| ***Hospital type: Primary Secondary Tertiary Specialized***  ***If Specialized, Specify: ………………………………………………*** | |
| ***Hospital Ownership: Public Faith-based Private NGO Unknown:…………………………………………………..*** | |
| ***OTHER HOSPITAL DETAILS***  *Does your hospital annual workplan include IPC activities? Yes No* | |
| *Does your IPC program have an annual workplan approved by the hospital administration? Yes No* | |
| *If yes, does your IPC annual workplan include HAI surveillance? Yes No* | |
| *Which HAI surveillance does your hospital participate in the previous year?* | |
| *SSI CLABSI/PLABSI CAUTI HAP/VAP None* | |
| *Is there a functional bacteriology lab to support HAI surveillance? Yes No* | |
| *Is hand hygiene compliance monitored in last 3months? Yes No*  *if Yes, what was the average hand hygiene compliance score for the hospital? ………………* | |

***DOES YOUR HOSPITAL HAVE THE FOLLOWING IN PLACE FOR HAI PREVENTION?***

|  | ***Guideline*** | ***Care bundle*** | ***Training*** | ***Checklist*** | ***Audit*** | ***Surveillance*** | ***Feedback*** |
| --- | --- | --- | --- | --- | --- | --- | --- |
| ***ICU/HDU/SCBU/ER*** | | | | | | | |
| ***HAP*** |  |  |  |  |  |  |  |
| ***VAP*** |  |  |  |  |  |  |  |
| ***Bloodstream infections*** |  |  |  |  |  |  |  |
| ***CLABSI*** |  |  |  |  |  |  |  |
| ***PLABSI*** |  |  |  |  |  |  |  |
| ***CAUTI*** |  |  |  |  |  |  |  |
| ***SSI*** |  |  |  |  |  |  |  |
| ***Antimicrobial use*** |  |  |  |  |  |  |  |
| ***HOSPITAL WIDE/ OTHER WARDS*** | | | | | | | |
| ***HAP/VAP*** |  |  |  |  |  |  |  |
| ***Bloodstream infections*** |  |  |  |  |  |  |  |
| ***CAUTI*** |  |  |  |  |  |  |  |
| ***SSI*** |  |  |  |  |  |  |  |
| ***Antimicrobial use*** |  |  |  |  |  |  |  |

**APPENDIX C**

**SIERRA LEONE NATIONAL HEALTHCARE ASSOCIATED INFECTIONS SURVEILLANCE**

***WARD FORM***

***(To be completed once for each ward)***

**WARD INDICATORS COLLECTED AT WARD LEVEL**

| **Ward Name:**  **……………………………………………….** | **Ward Type:**  Medical [ ] Surgical [ ] Pediatric [ ] ICU/HDU [ ] Maternity [ ] Emergency [ ] Others:…………………………… |
| --- | --- |
| **Hospital code:………………………..** | **^$^Ward Code: …………………………….** |
| ***Ward total Patients:……………….** | **^#^Ward eligible patients: ……………** |
| **Ward Survey Date:………………….**  **Ward Investigator(s):**  1.  2.  3.  4.  5. | |

| **Indicator** | **Number** |
| --- | --- |
| Number of beds with ABHR at point of care |  |
| Number of hand washing stations in wards |  |
| Number of hand hygiene observations done in last 3 months |  |
| Number of patient rooms in ward |  |
| Number of single patient rooms in ward |  |
| Number of single patient rooms with individual toilet and shower in ward |  |

^$^Ward Code: Hospital code + abbreviation of ward name (eg Ward 1 at Connaught hospital could be CHW1)

**Note: Ward Total Patients also includes day cases and/or long-term care patient*

^#^Ward eligible patients – Patient >1 month of age admitted in the ward on or before 8am today and is not already discharged home.

**APPENDIX D**

**SIERRA LEONE NATIONAL HEALTHCARE ASSOCIATED INFECTIONS SURVEILLANCE**

**PATIENT FORM**

| **SECTION A** | **GENERAL DETAILS** | | | |
| --- | --- | --- | --- | --- |
| Serial number: ……… Name of hospital: …………………………………..…. Patient hospital ID: ………… | | | | |
| Patient Code: …………………... Hospital Code :………………………. Ward Code:…………………… | | | | |
| **SECTION B** | | **DEMOGRAPHIC INFORMATION** | | |
| Age: | | Gender: Male [ ] Female [ ] | | |
| **SECTION C** | | | **RISK FCACTORS** | |
| **Major risk factors** | | | Smoking [ ] Diabetes [ ] Stroke/paraplegia [ ] Chronic lung disease [ ] Cancer [ ] Obesity [ ] Malnutrition [ ] Leukopenia [ ] HIV [ ] Burns [ ] Immobility [ ] Loss of consciousness [ ] | |
| **Antibiotic use since admission** | | | None [ ] Prophylaxis [ ] Treatment [ ] | |
| **Other risk factors:** | | | Surgical drain [ ] Steroid use [ ] Blood transfusion [ ] Use of oxygen devices [ ] None [ ] | |
| **Triger for CLABSI/ PLABSI;**  **Intravascular catheter (**V or central line) was in place for >2 calendar days during this preset admission | | | Yes, and catheter is still in place [ ]  Yes, but catheter was removed in within the past 24hrs [ ]  No, catheter was never in place or has been removed for more than 24hr [ ]  **If yes,** Peripheral [ ], Duration (days) the IV catheter has/was in place……………  Central [ ] , Duration (days) the central catheter has/was in place ………… | |
| **Triger for CAUTI; Indwelling catheter** was in place for >2 calendar days during this preset admission | | | Yes, and indwelling catheter is still in place [ ]  Yes, but an indwelling catheter was removed in within the past 24hrs [ ]  No, indwelling catheter was never in place or has been removed for more than 24hr [ ]  **If yes,** Urethral closed [ ], Duration (days) the ureteral catheter has/was in place…………  Suprapubic [ ], Duration (days) the Suprapubic catheter has/was in place ………… | |
| **Triger for HAP/VAP;**  Patients received mechanical ventilation or with history of previous hospitalization for >2 calendar days | | | Yes, and mechanical ventilation is still in place [ ]  Yes, but Mechanical ventilation in ward/ICU/theatre was removed in within the past 24hrs [ ]  No mechanical ventilation but, use of oxygen devices [ ] …………………………  **If yes,** Duration (days) the mechanical ventilation has/was in place…………  Duration (days) the use of oxygen devices has/was in place ………… | |
| **Triger for SSI;**  **Surgery in past 30 days** | | | Yes [ ] No [ ]  **If yes,** Elective [ ] Emergency [ ] Number of days since surgery: ………..  Name of surgical procedure: …………………………………………….……...…..  Classification of surgical wound: Clean [ ] Clean-contaminated [ ] Contaminated [ ] Dirty [ ]  **ASA Score*:** I [ ] II [ ] III [ ] IV [ ] V [ ] | |
| **Did any of the HAI trigger apply for this patient** | | | Yes [ ] No [ ] **If Yes,** select which HAI(s) below and continue with Section D;  CLABSI/PLABSI [ ] CAUTI [ ] HAP/VAP [ ] SSI [ ]  **If No** end the survey for this patient | |
|  | | |  | |
| **SECTION D** | | | **CLINICAL AND LABORATORY FEATURES/CRITERIA** | |
| **CLABSI/PLABSI**  . | | | 1. Laboratory-confirmed bloodstream infection with recognized pathogen cultured from 1 or more blood samples [ ] 2. Fever [ ] Chills [ ] Hypotension [ ] (For patients ≤1 year hypothermia [ ] Apnoea [ ] Bradycardia [ ] ) 3. Common skin contaminant is cultured from 2 or more blood cultures drawn on separate occasions [ ] 4. No apparent infection at another site [ ] or Clinician institutes treatment for sepsis [ ] | Patient have only criteria 1, or have at least one parameter of each 2&3, record as **Microbiologically confirmed CLABSI/PLABSI [ ]**  Patient have at least one parameter of criteria 2 and criteria 4, record as **Microbiologically unconfirmed CLABSI/PLABSI [ ]** |
| **CAUTI** | | | 1. Frequency [ ] Urgency [ ] Flank/suprapubic pain or tenderness [ ] Fever (>38^o^C) [ ] Altered mental status in elderly (>70 years) [ ] In children: a crying baby when passing urine [ ] 2. Positive urine culture [≥10^5^ microorganisms (≤2 species)/ml] [ ] 3. Positive dipstick (leucocyte/nitrite) [ ] Pyuria (>10 WBC/ml) [ ] Organisms/gram of unspun urine [ ] ≥2 urine cultures same uropathogen ≥10^2^ organisms/ml [ ] *Clinician diagnosis of UTI [ ] Clinician treatment of UTI [ ]* | Patient meet at least one parameter of criteria 1, and criteria 2, record as M**icrobiologically confirmed CAUTI [ ]**  Patient must meet at least two parameters of criteria 1, and one parameter of criteria 3, record as **Microbiologically unconfirmed CAUTI [ ]** |
| **HAP/VAP** | | | 1. Fever [ ] Leukopenia (<4000 WBC/mm3) or Leucocytosis (≥12,000) [ ], 2. New onset or change in character of sputum, or increased respiratory secretions, or increase suctioning requirements [ ]; Cough [ ]; Dyspnoea [ ]; Tachypnoea [ ] Rales/bronchial breathing [ ]; Worsening gas exchange [ ] 3. One chest X-ray with at least New or progressive and persistent infiltrates [ ]; consolidation [ ] or Cavitation [ ] 4. Positive cultures in the blood or respiratory secretions or pleural fluid or histopathologic specimen [ ] | Patients meet at least one parameter of each 1,2 and 3 or 4, record as **Radiologically confirmed HAP or VAP [ ]**  Patients meet at least one parameter of each 1 and 2, record as **Radiologically unconfirmed HAP or VAP [ ]** |
| **SSI** | | | Surgical site infection: Yes [ ] No [ ]   1. A purulent (pus) discharge in, or coming from the wound (including evidence of an abscess) [ ] **or** 2. Evidence of fever with painful, spreading erythema surrounding the surgical site [ ] **or** 3. Any reopening of the surgical wound [ ]   If yes, type:  Date documented: | **Superficial SSI [ ]**  **Deep SSI [ ]**  **Organ space [ ]** |
